# NR5A2 as a potential target for exercise to improve metabolic syndrome

**DOI:** 10.18632/aging.204606

**Published:** 2023-03-26

**Authors:** Lingxiu Meng, Fusheng Dong, Junguo Deng

**Affiliations:** 1 Department of Cardiology, Qinhuangdao Second Hospital, Qinhuangdao, Hebei 066600, PR China; 2 Department of Anesthesiology, Qinhuangdao Second Hospital, Qinhuangdao, Hebei 066600, PR China

**Keywords:** metabolic syndrome, movement, NR5A2, potential targets, cardiovascular diseases

## Abstract

Background: Metabolic syndrome is a syndrome of a variety of metabolic disorders. Exercise is beneficial to the human body. However, the association of NR5A2 and exercise with metabolic syndrome remains unclear.

Methods: Download the GSE10540 and GSE12385 from GEO database. Bioinformatics analysis was used to screen the hub molecular of the metabolic syndrome. Forty 3-week-old C57BL/6J male mice were used in this study. The mean body weight was (17.5 ± 2.1) g. After 10 days of adaptive feeding, they were randomly divided into 4 groups according to the random number table method: Model + Exercise (*n* = 10), Model (*n* = 10), Model/NR5A2-OE (*n* = 10), Model/NR5A2-KO (*n* = 10). Western Blotting was performed to detect the expression of hub genes and signaling pathway.

Results: There were 349 DEGs in GSE10540 and 49 DEGs in GSE12385. 10 core genes were obtained. GO showed that differentially expressed genes were mainly enriched in vascular morphogenesis, contractile fiber fraction, chemotaxis, and MAPK cascade regulation. KEGG showed that MAPK signaling pathway was a significant section in the metabolic syndrome. PIK3R2, STRA8, FLT1, DMRT1, FGF22, NR5A2, and FLT were up-regulated and PRDM14, POU5F1, and KDR were down-regulated in metabolic syndrome after exercise.

Conclusion: The expression of NR5A2 is down-regulated in metabolic syndrome, and exercise can increase the expression level of NR5A2. NR5A2 might be used as a potential target for exercise to improve metabolic syndrome.

## INTRODUCTION

Metabolic syndrome is a risk factor leading to diabetes and cardiovascular diseases [1]. Metabolic syndrome is not infectious, and its incidence in China is 14–16%, and it has been increasing in recent years. It is more common in obesity, hypertension and hyperglycemia. The risk of disease will increase with age, accompanied by complications [2, 3]. Metabolic syndrome can lead to obesity or being overweight, dyslipidemia, hypertension, and promote the occurrence of atherosclerotic cardiovascular disease [4]. Due to obesity, patients with metabolic syndrome often have slow movement and decreased physical strength. Some people need long-term medication, which can easily cause psychological disorders. Most patients can delay the disease process by active treatment, but it is difficult to cure and can relapse [5]. However, the etiology of metabolic syndrome is unclear, and its possible genetic factors might induce metabolic syndrome.

Exercise can promote metabolism, promote the burning of excess fat in the body, help individuals stay in shape and lose weight, increase muscle content, promote bone growth, improve sleep quality, increase vital capacity, benefit the heart, enhance disease resistance, release anxiety, and regulate psychological state [6, 7]. The transcriptional changes induced by exercise may provide new mechanistic insights in the field of improving metabolic syndrome through exercise. However, the reason why exercise improves metabolic syndrome is not clear.

Bioinformatics technology is an interdisciplinary subject of biology and computer. It uses modern information technology to simulate and predict protein structure based on the analysis of a large number of data such as DNA sequencing and functional genome, and is used for drug design [8, 9].

NR5A2 (nuclear receptor subfamily 5 group A member 2) is A protein-coding gene and A lone nuclear receptor that requires ligand-activated transcription factors [10]. This encoded protein is involved in the expression of cholesterol biosynthesis genes and may be an important regulator of embryonic development [11, 12]. However, the relationship between NR5A2 and exercise in improving metabolic syndrome is still unclear.

Therefore, the study aimed to use bioinformatics to explore core genes of metabolic syndrome between before and after exercise, and animal experiments were used to verify the value of NR5A2 in the exercise curing metabolic syndrome.

## METHODS

### Exercise metabolic syndrome dataset

The movement of metabolic syndrome GSE10540 data sets and the configuration file generated from using GPL2986 GEO (http://www.ncbi.nlm.nih.gov/geo/) to download, including five sports blood samples before and after five movement blood samples. GSE12385 and configuration files generated from the use of GPL4133 to download, including 8 volunteers before and after exercise PBMC. Differentially expressed genes (DEGs) were used to identify exertional metabolic syndrome.

### Screening of DEGs

The R package (https://cloud.r-project.org/) “limma” was used to perform probe summarization and background correction for the matrices of GSE10540 and GSE12385, respectively. Matrix score, immune score and disease were used as the grouping basis for differential gene screening. The cutoff criterion for DEG was FDR <0.05. Volcano diagram was made.

### Weighted gene co-expression network analysis (WGCNA)

Using the GSE10540 matrix, WGCNA (https://horvath.genetics.ucla.edu/html/CoexpressionNetwork/Rpackages/WGCNA/index.html) can identify gene sets with similar expression patterns, analyze the associations between gene sets and sample phenotypes, and draw regulatory networks between gene sets, and identify hub genes.

Firstly, the gene co-expression network is constructed. Usually, the expression pattern between two genes is used to calculate a correlation coefficient between them, and then the gene network is constructed based on the correlation coefficient. After establishing the relationship between genes, threshold values were used to classify those genes that were closely related, and we divided the closely related genes into a module. Do some feature analysis on module, including assigning eigenvalues to it and GO enrichment of genes in module to explore its function. The key modules related to biological problems are selected based on module expression pattern and module functions. Internal genes of key modules were analyzed, including the function of internal annotated genes and a relationship between their regulatory levels to identify some key regulatory genes in the module.

### Construction and analysis of protein-protein interaction (PPI) network

STRING (https://cn.string-db.org/cgi/input.pl) was used to collect, scoring and integrate all publicly available protein sources - protein interaction, and predicted by calculation to complement these sources. In this study, the list of differential genes was input into the STRING to construct a PPI for predicting core genes (confidence >0.4). Cytoscape (https://cytoscape.org/) could provide visualization. The module with the best correlation was found by MCODE. Two algorithms (MCC and DMNC) were used to calculate the ten best correlation genes and take the intersection, and the core gene list was derived after visualization.

### Functional enrichment analysis

GO and KEGG analysis are computational methods to evaluate gene functions and biological pathways. This study will Wayne figure out the difference of gene list input KEGG. The R package org.Hs.eg.db (version 3.1.0) was used for the GO annotation.

In addition, we use the Metascape database (http://metascape.org/gp/index.html), for the above differences in gene enrichment of function analysis and export list.

### GSEA analysis

Gene set enrichment analysis (GSEA) (http://www.gsea-msigdb.org/gsea/index.jsp) was used to perform GO and KEGG analyses on complete genomes. In our study, whole blood samples were grouped by pre-exercise and post-exercise, and whole-genome GO and KEGG analyses were performed. Developed by GSEA.

### Heat map of gene expression

The R package heatmap was used to make a heatmap of the expression of core genes found by the two algorithms in the PPI network in GSE10540 and GSE12385, and to visualize the expression differences of core genes between the whole blood samples before and after exercise.

### CTD analysis

We find from CTD website (http://ctdbase.org/) and core genes differentially expressed most related disease, before selecting eight diseases with EXCEL visual representation.

### Establishment of metabolic syndrome animal model

Forty 3-week-old C57BL/6J male mice were used in this study. The mean body weight was (17.5 ± 2.1) g. After 10 days of adaptive feeding, they were randomly divided into 4 groups according to the random number table method: Model + Exercise (*n* = 10), Model (*n* = 10), Model/NR5A2-OE (*n* = 10), Model/NR5A2-KO (*n* = 10). All mice were fed with high-fat purified diet and high-sugar diet. The temperature of the animal house was 21–23 degrees and the relative humidity was 40%, and the animals were fed AD libitum with water. Fresh feed was changed daily, and food intake and water intake were monitored. High fat purified feed high sugar feed was produced by Huafukang Company.

### Western blotting

The collected blood was used to extract protein, sample loading, electrophoresis, membrane transfer, and 5% skim milk blocking for 2 h. The primary antibody diluted 1:1000 was incubated overnight at 4°C. After incubation with 1:5000 diluted secondary antibody (2 h, 4°C), ECL A solution and ECL B solution were mixed in medium volume in A centrifuge tube in A dark room. Double PE gloves or other transparent films were attached to the exposure box. The image J was used to analyze the photo. ACTIN is taken as the internal parameter. The NR5A2 antibody (22460-1-AP, Proteintech, USA) was used to detect the expression of NR5A2. Goat Anti-Rabbit IgG H&L (HRP)-labeled second antibodies (ab205718, Abcam, USA) of the corresponding species (dilution rate = 1:5000) were then incubated at room temperature for 30 min.

### Statistics

All data were analyzed using GraphPad Prism 8.0 software. Experimental results were presented as mean ± SD. Students’ *T* test or Fisher test were used for comparison. Statistically significant is defined as *P* < 0.05.

### Availability of data and materials

The datasets used and/or analyzed during the current study are available from the corresponding author on reasonable request.

## RESULTS

### Detection of differentially expressed genes (DEGs)

**Figure 1:**
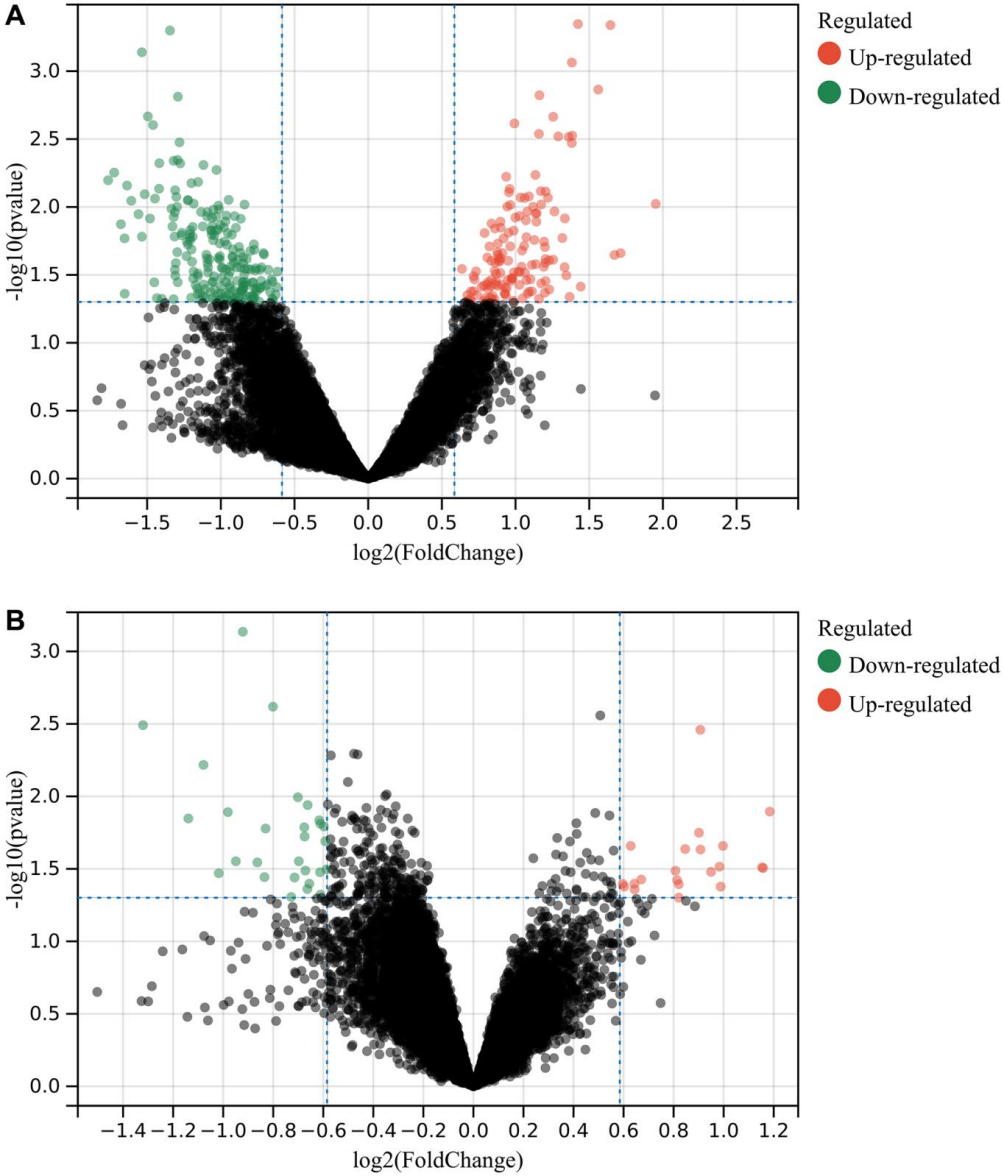
**Detection of differentially expressed genes (DEGs).** (**A**) 349 DEGs in GSE10540 (**B**) 49 DEGs in GSE12385.

In this study, DEGs were identified according to the matrix of GSE10540 and GSE12385, and the intersection was taken by Venn diagram. There were 349 DEGs in GSE10540 (Figure 1A) and 49 DEGs in GSE12385 (Figure 1B).

### WGCNA analysis

**Figure 2:**
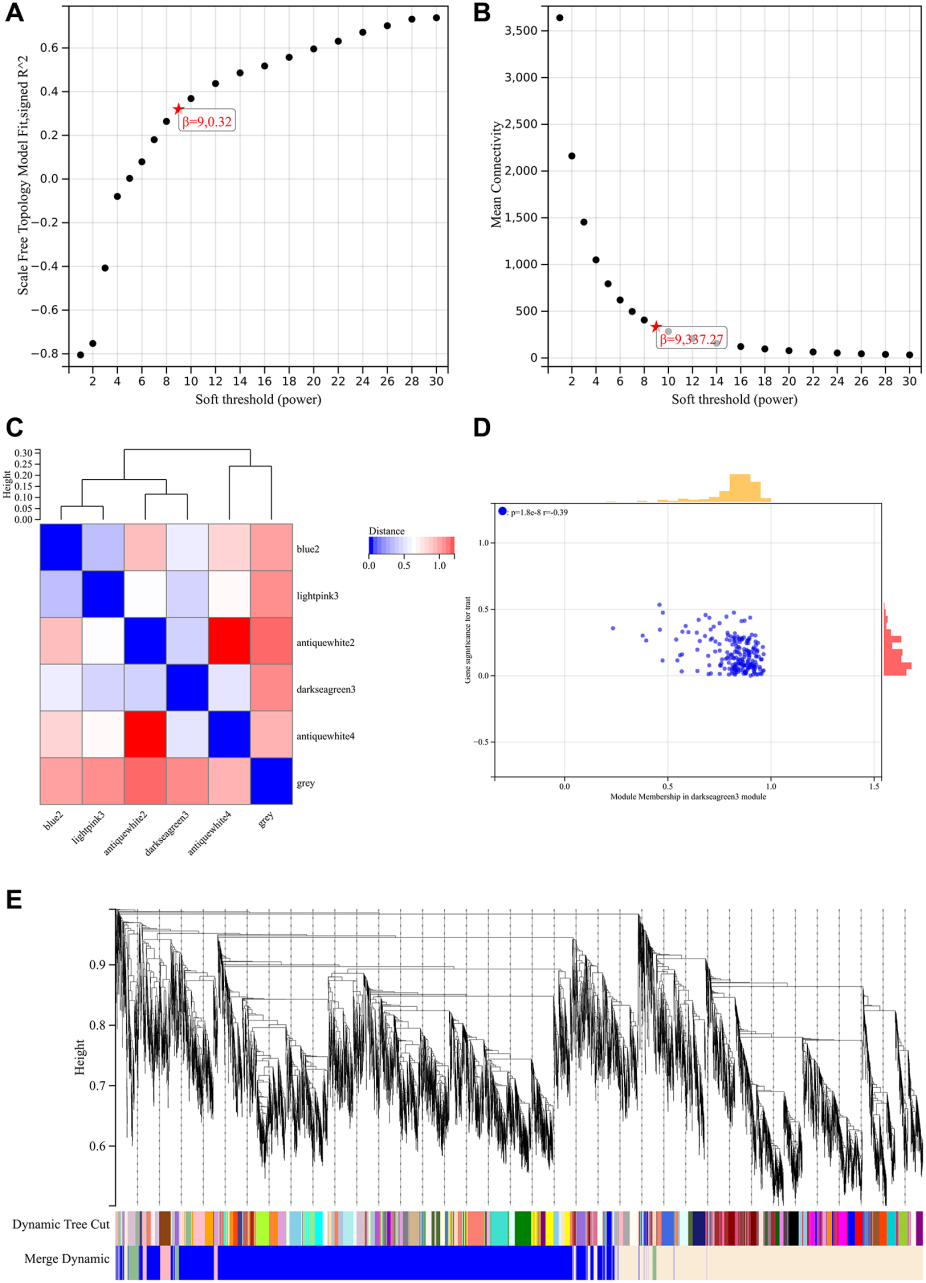
**WGCNA analysis.** (**A**) β = 9, 0.32 (**B**) β = 9, 337.27 (**C**) Hierarchical clustering tree of all genes and six significant modules were generated. (**D**) Darkseagreen3 module had the highest negative correlation with the status of exercise metabolic syndrome (**E**) There is a high degree of independence between modules.

The soft threshold power was 9 (Figure 2A, 2B). Hierarchical clustering trees were constructed and six modules were presented (Figure 2C). Dendrograms and heatmaps of genes showed no significant differences in interactions between different modules, indicating a high degree of independence between these modules (Figure 2E). The Darkseagreen3 module was most negatively correlated with the status of the exertional metabolic syndrome (Figure 2D). In addition, we calculated the module characteristic vector correlation with the expression of genes for MM, by tagging based on MM (|MM| > 0.8), a total of 147 genes was identified as gene and clinical representation is closely related to the core.

### PPI network

**Figure 3:**
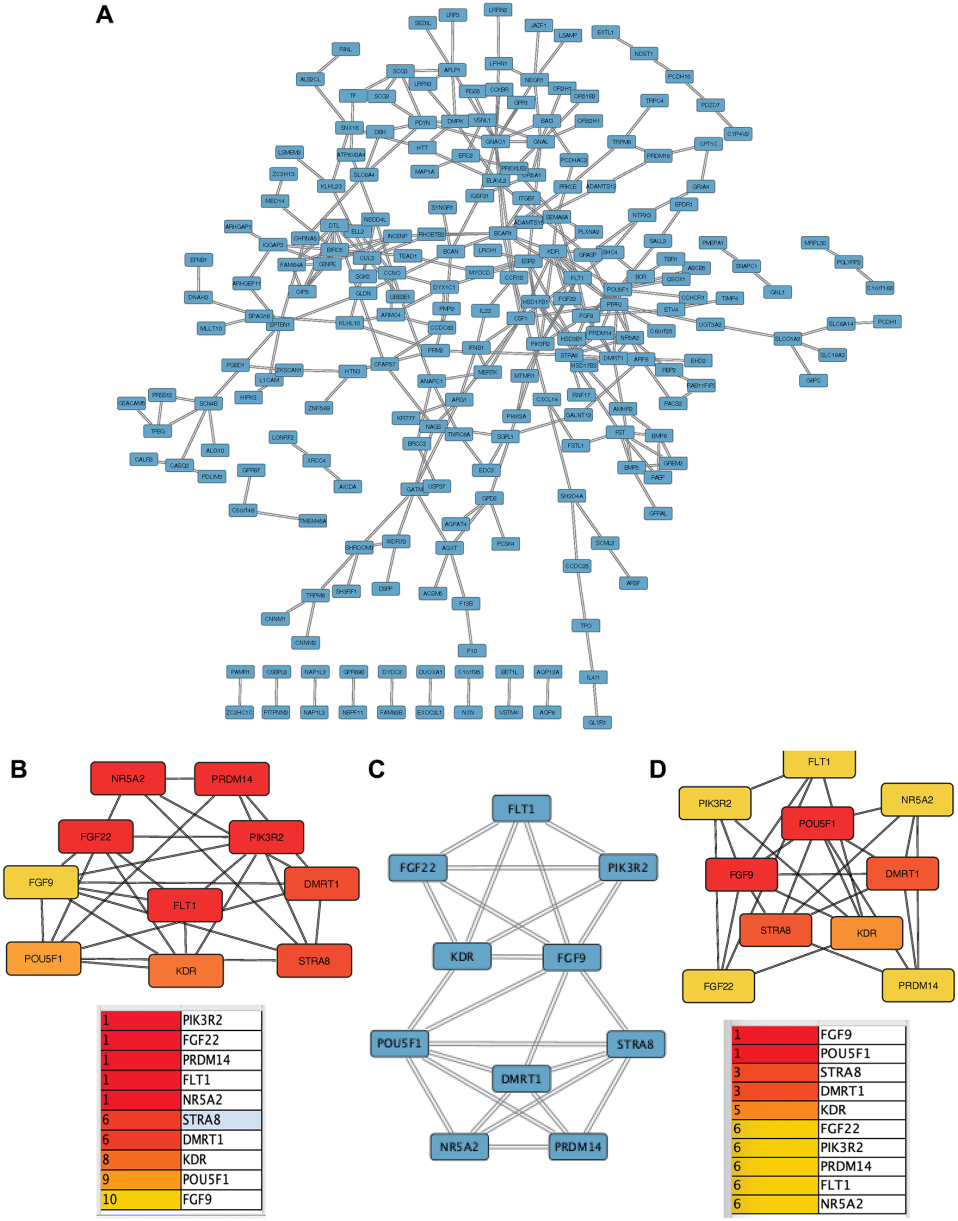
**Construction and analysis of protein-protein interaction (PPI) network.** (**A**) PPI network of DEGs. (**B**) MCC algorithm. (**C**) MCODE calculates the core module. (**D**) DMNC algorithm.

349 predicted DEGs were identified in the PPI network (Figure 3A). Hub genes were identified by the MCC algorithm (Figure 3B). We used MCODE to calculate the core module (Figure 3C). Hub genes were identified by the DMNC algorithm (Figure 3D). A total of 10 core genes (PIK3R2, STRA8, FLT1, DMRT1, FGF22, NR5A2, FLT, PRDM14, POU5F1, KDR) were obtained.

### Functional enrichment analysis

**Figure 4:**
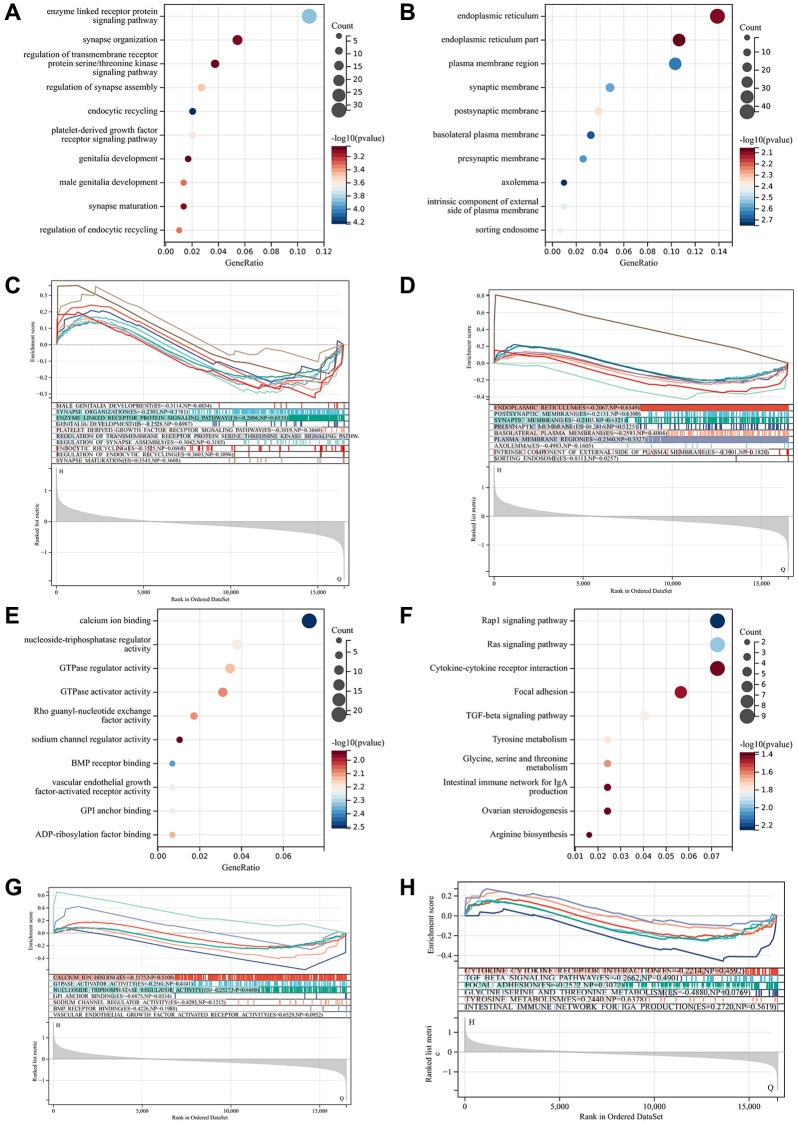
**Functional enrichment analysis and GSEA analysis.** (**A**) Biological process by bubble diagram. (**B**) Cell component by bubble diagram. (**C**) Biological process by GSEA. (**D**) Cell component by GSEA. (**E**) Molecular function by bubble diagram. (**F**) Kyoto Encyclopedia of Genes and Genomes by bubble diagram. (**G**) Molecular function by GSEA. (**H**) Kyoto Encyclopedia of Genes and Genomes by GSEA.

GO showed that the DEGs were mainly enriched in vascular morphogenesis, chemotaxis, and MAPK cascade regulation. KEGG analysis showed DEGs were mainly enriched in MAPK signaling pathway, atherosclerosis, and cytofactor-cytokine receptor interaction. The *p*-values for GO terms are shown in Figure 4A, 4B, and 4E. The *P* values for KEGG terms are shown in Figure 4F. These may improve the biological understanding of these genes.

### GSEA analysis

In this study, we used the GSE10540 whole gene expression matrix for enrichment analysis, which showed that the GSEA enrichment projects were verified with the GO and KEGG enrichment projects in the differentially expressed genes (Figure 4C, 4D, 4G, 4H).

### Enrichment analysis by Metascape

**Figure 5:**
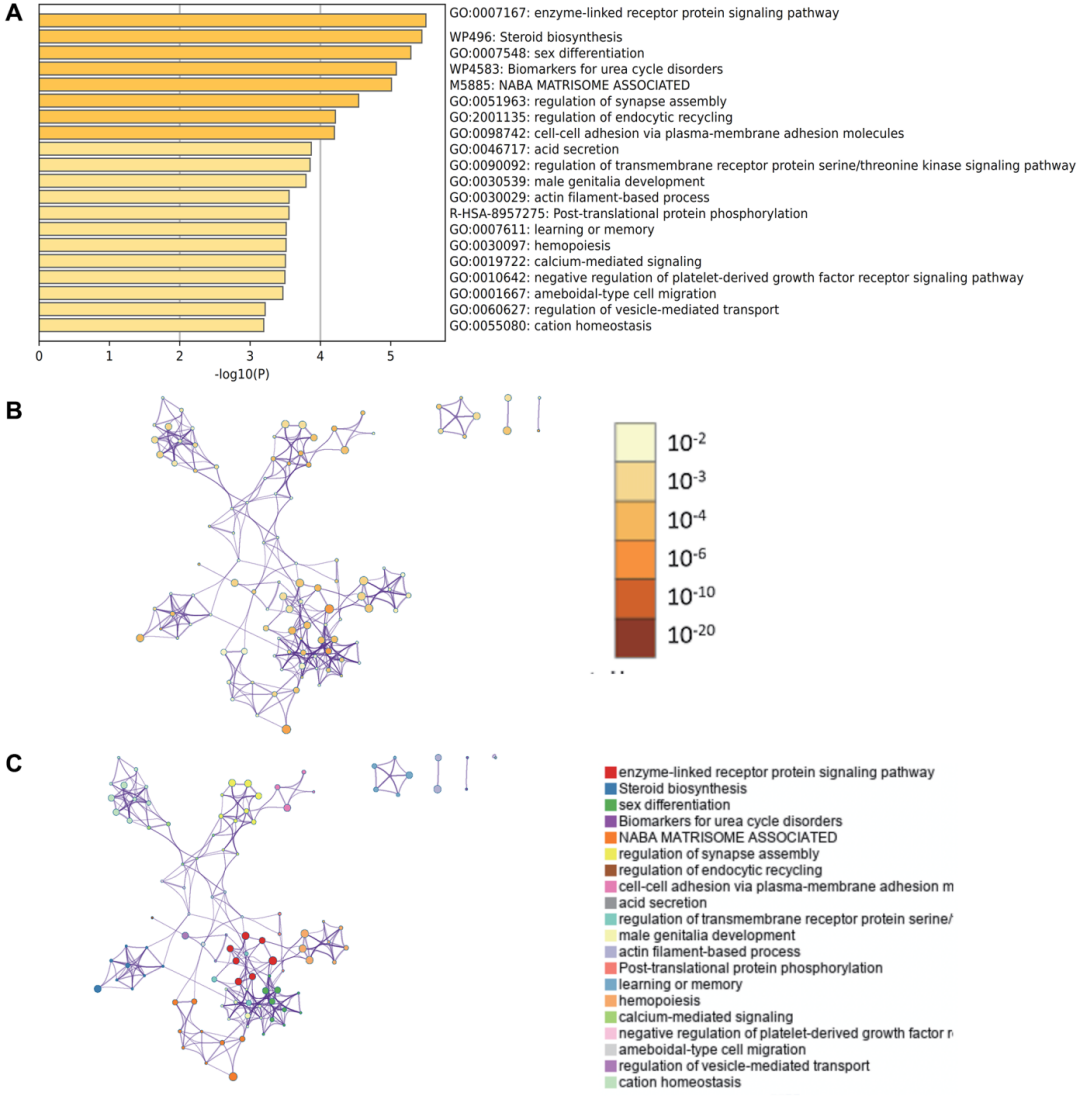
**Enrichment analysis by Metascape.** (**A**) GO enrichment terms (**B**) enrichment networks colored by enrichment terms (**C**) have enrichment networks colored by *p*-values.

**Figure 6:**
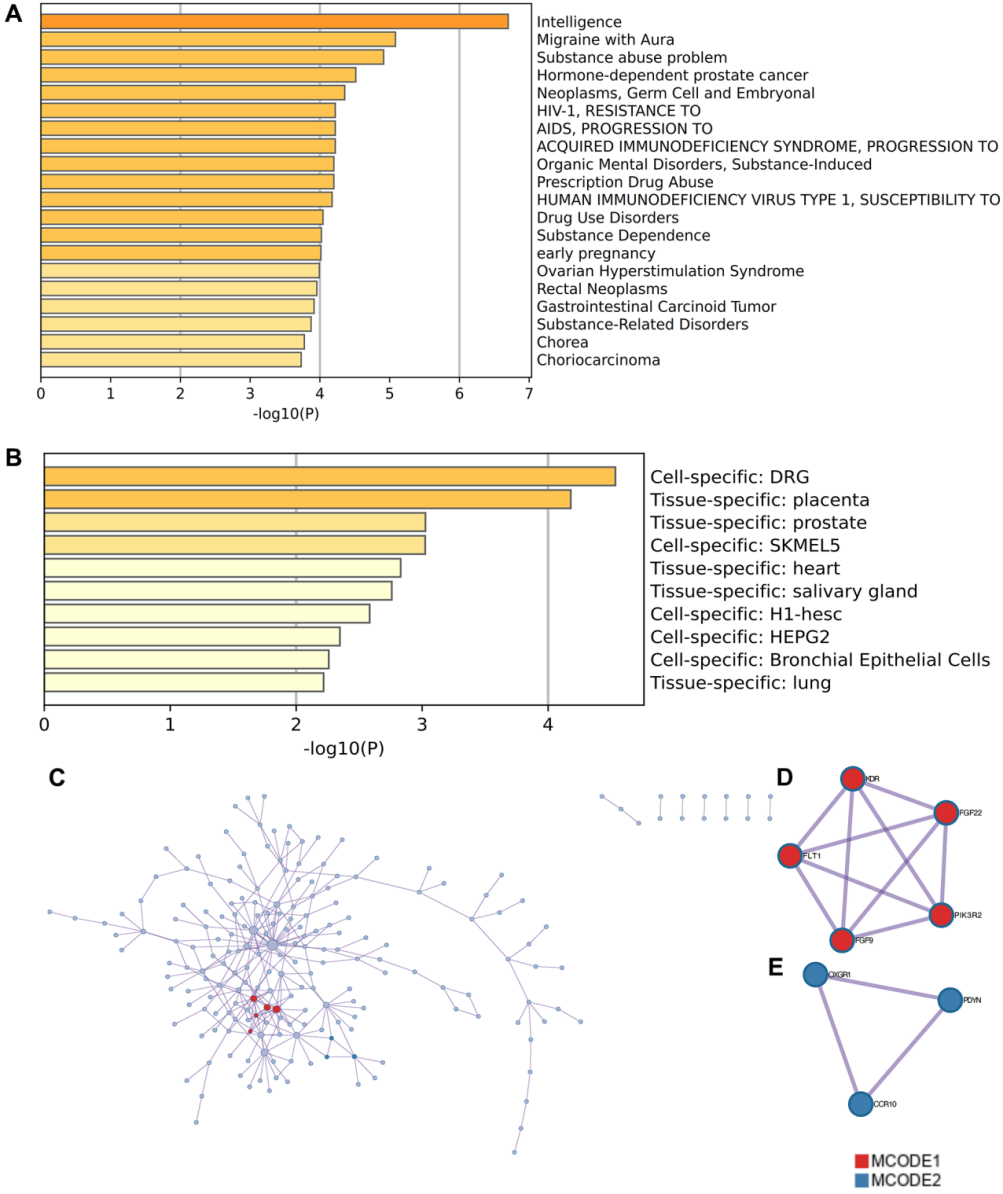
**Enrichment analysis by Metascape.** (**A**) modules about enriched disease terms (**B**) enriched terms about enriched cell species (**C**) PPI network (**D**) MCODE1 (**E**) MCODE2.

The content enriched by Metascape included GO enrichment terms (Figure 5A) and had enrichment networks colored by enrichment terms and *p*-values (Figure 5B, 5C). It also includes a module for enriching disease terms (Figure 6A), an enrichment term for enriching cell types (Figure 6B), and a PPI network and core module formed in Metascape website based on core genes (Figure 6C–6E).

### CTD analysis

**Figure 7:**
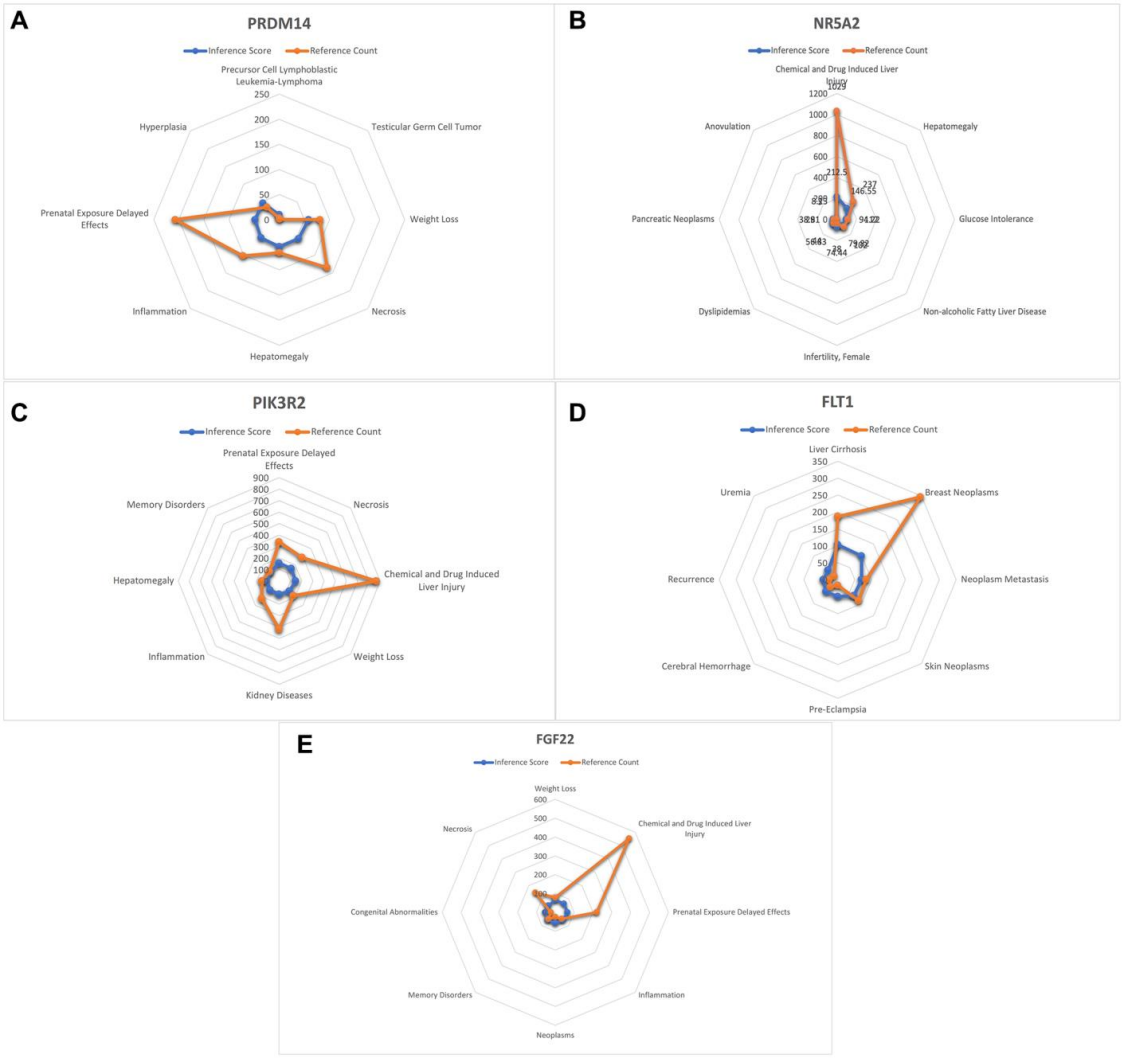
**Analysis of CTD.** (**A**) PRDM14 (**B**) NR5A2 (**C**) PIK3R2 (**D**) FLT1 (**E**) FGF22.

**Figure 8:**
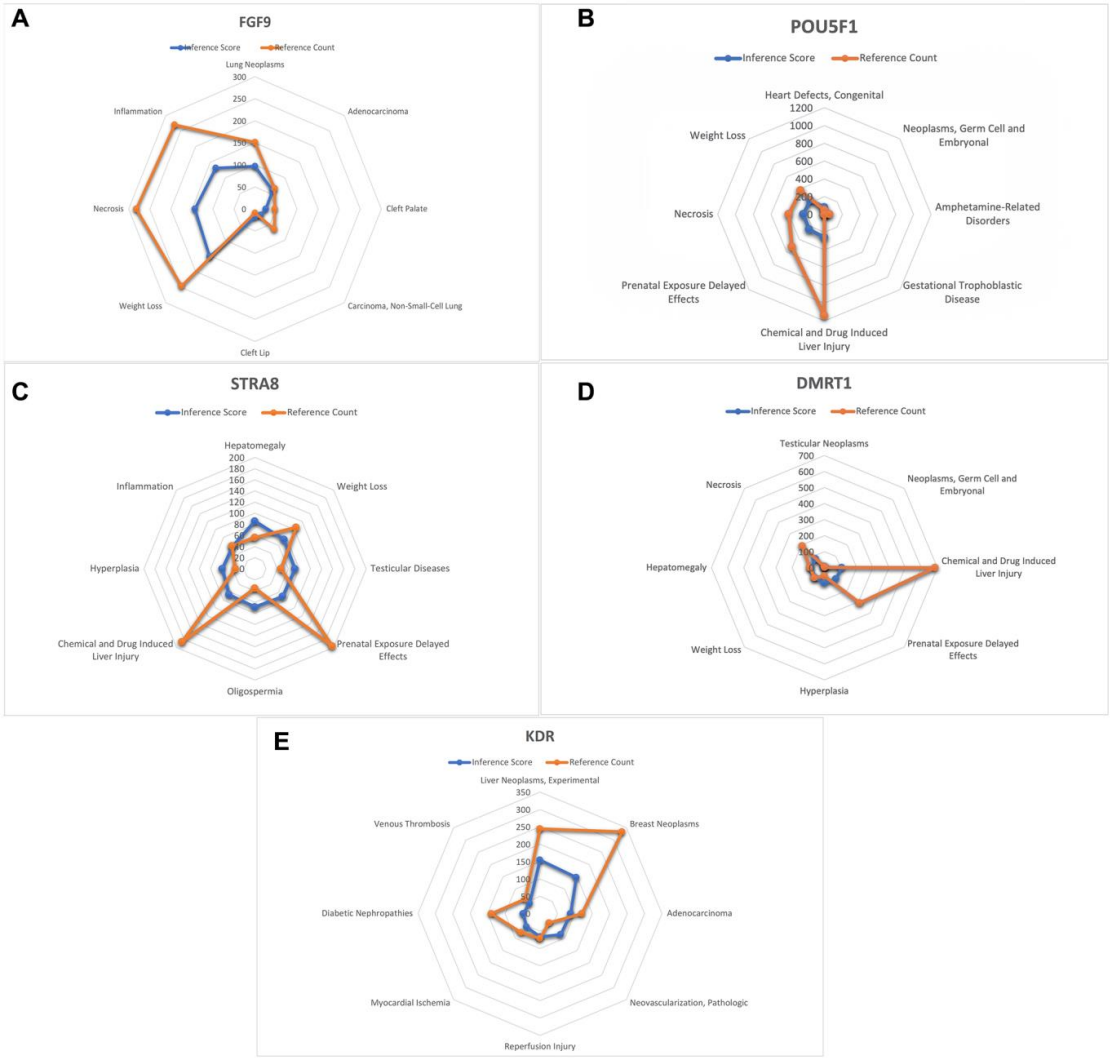
**Analysis of CTD.** (**A**) FGF9 (**B**) POU5F1 (**C**) STRA8 (**D**) DMRT1 (**E**) KDR.

In this study, we input the list of core genes into the CTD website to find diseases related to core genes and improve the understanding of gene-disease association (Figures 7 and 8).

### miRNA analysis

In this study, we input the hub gene list into Targetsacan to find relevant miRNA and improve the understanding of gene expression regulation. NR5A2 was related with the micRNA of hsa-miR-10a-5p and hsa-miR-10b-5p (Table 1).

**Table 1:** The prediction of micRNA for hub genes.

**Gene name**	**micRNA**		
**PIK3R2**	hsa-miR-30c-5p	hsa-miR-30b-5p	
**FGF22**	hsa-miR-6746-3p	hsa-miR-6827-5p	
**PRDM14**	None		
**FLT1**	hsa-miR-200c-3p	hsa-miR-429	hsa-miR-200b-3p
**NR5A2**	hsa-miR-10a-5p	hsa-miR-10b-5p	
**STRA8**	None		
**DMRT1**	None		
**KDR**	hsa-miR-424-5p	hsa-miR-15b-5p	
**POU5F1**	hsa-miR-335-5p		
**FGF9**	hsa-miR-140-5p		

### Heat map

**Figure 9:**
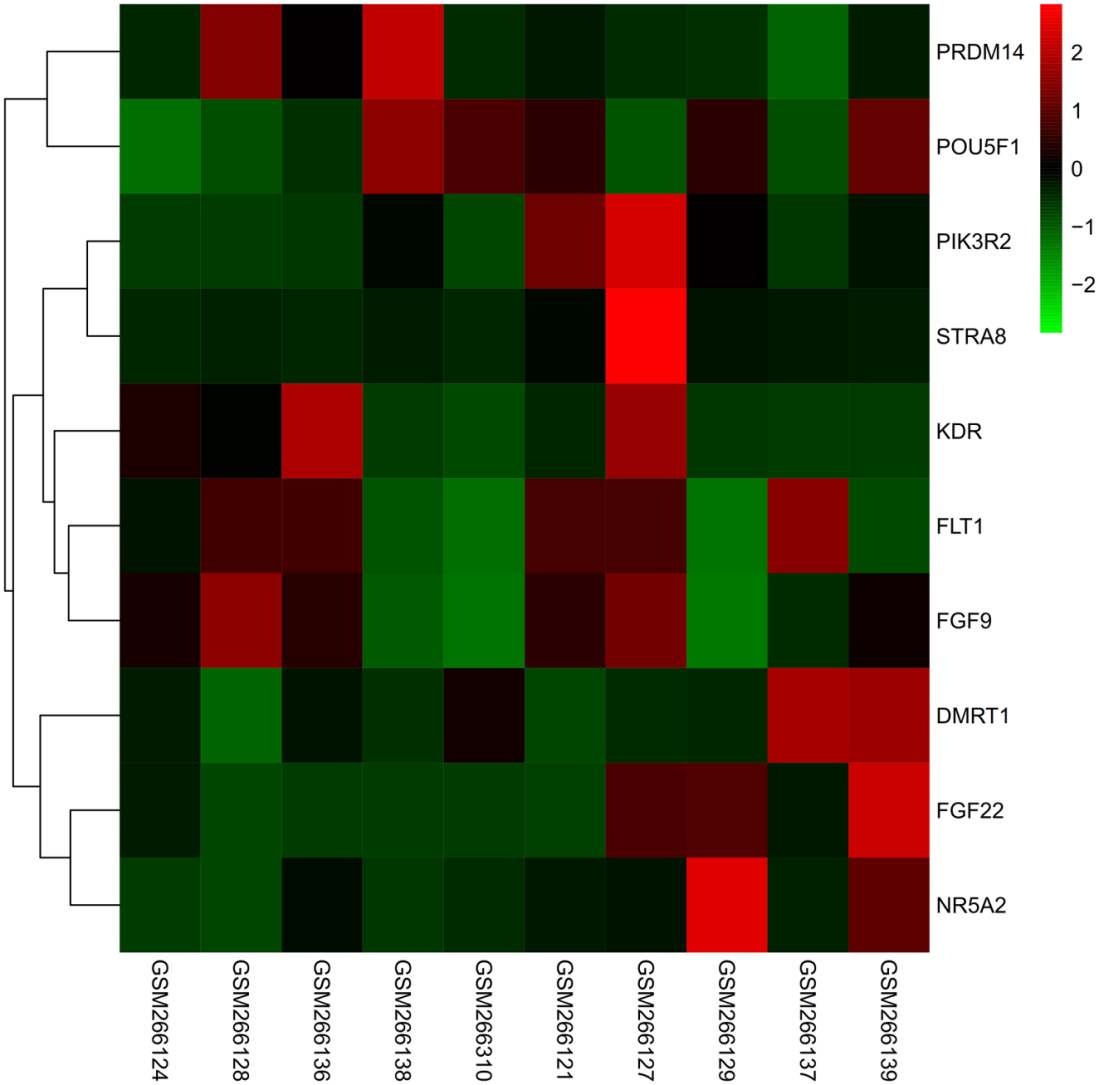
The expression of core genes in GSE10540 matrix was processed by heat map. PIK3R2, STRA8, FLT1, DMRT1, FGF22, NR5A2, FLT genes were up-regulated after exercise in metabolic syndrome, and PRDM14, POU5F1, KDR genes were down-regulated after exercise.

The expression of core genes in GSE10540 matrix was processed by heat map. PIK3R2, STRA8, FLT1, DMRT1, FGF22, NR5A2, and FLT genes were up-regulated in metabolic syndrome after exercise. PRDM14, POU5F1, and KDR were down-regulated after exercise (Figure 9).

### Western blot (WB)

**Figure 10:**
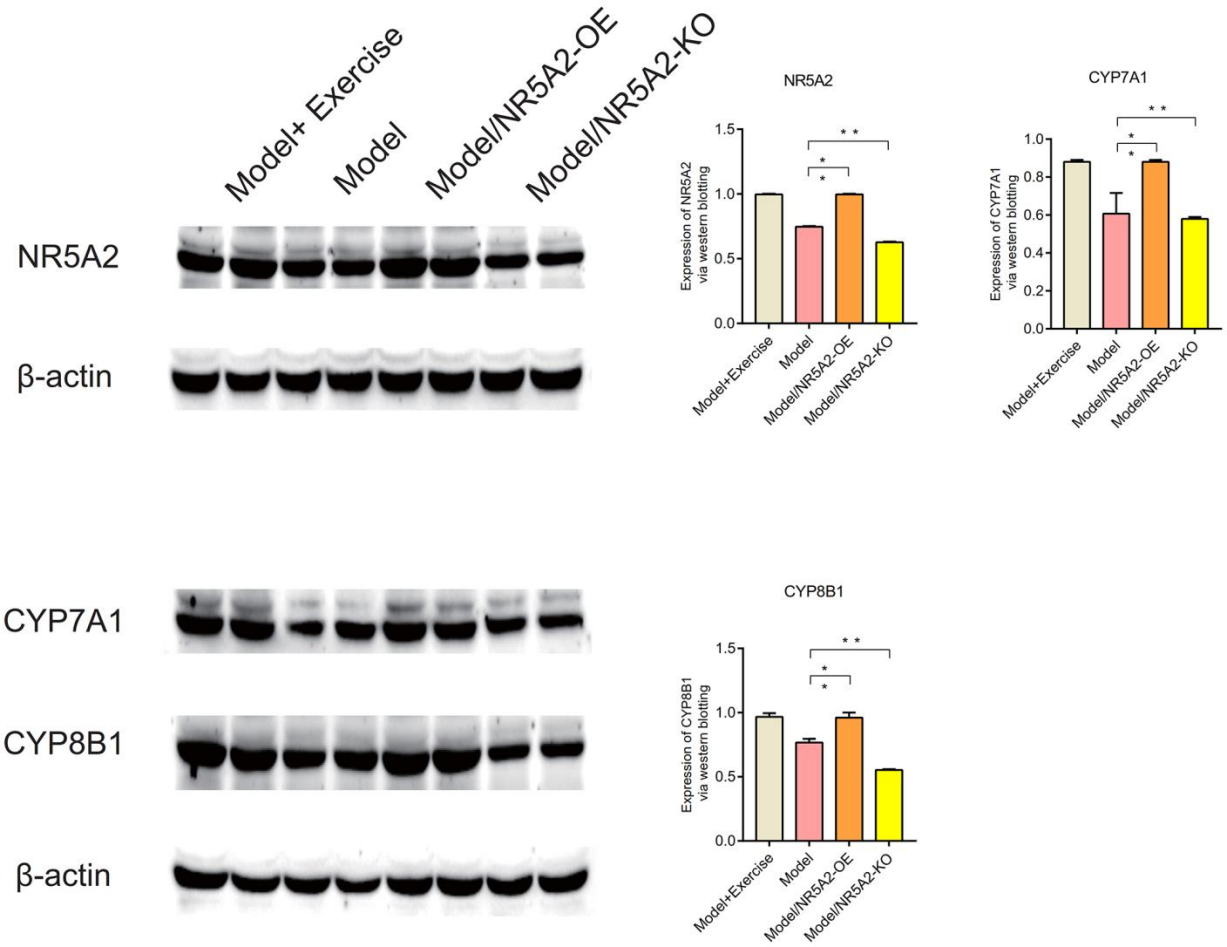
**Western blot (WB).** The expression levels of NR5A2, CYP7A1, and CYP8B1were up-regulated after exercise (*P* < 0.05).

**Figure 11:**
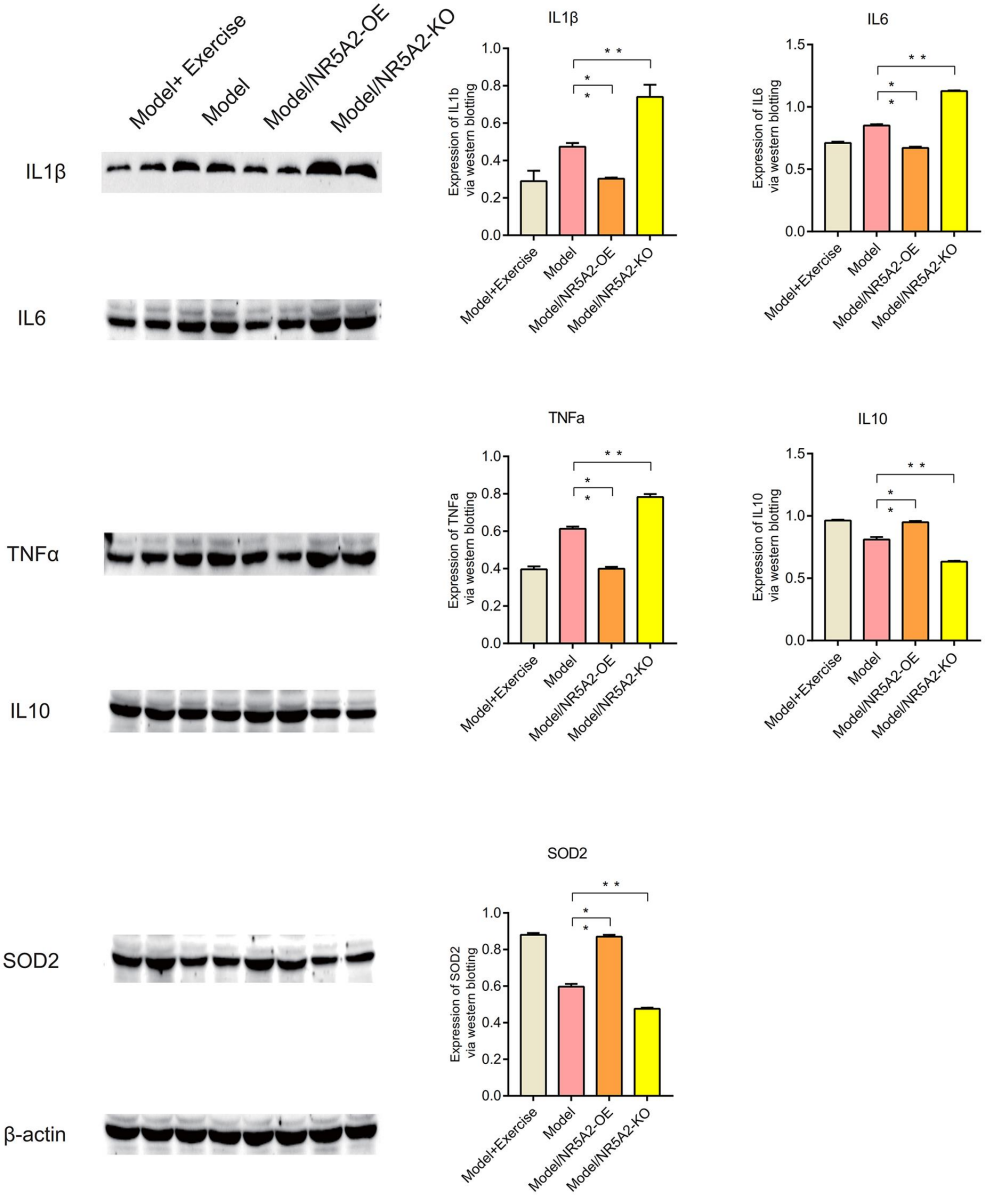
**Western blot (WB).** The expression levels of IL1β, IL6 and TNFa are down-regulated after exercise. IL10 and SOD2 were up-regulated after exercise (*P* < 0.05).

**Figure 12:**
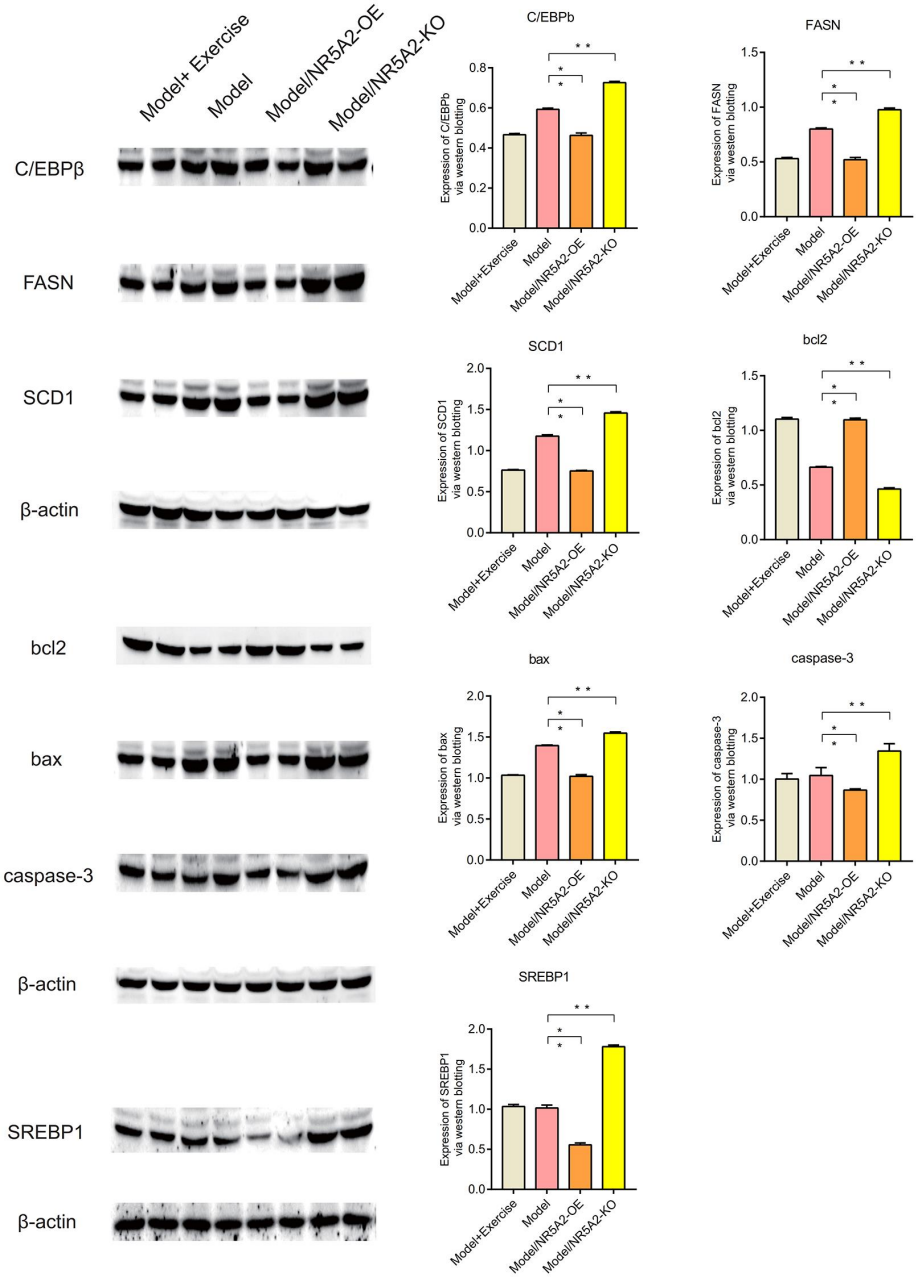
**Western blot (WB).** The expression levels of C/EBPb, FASN, SCD1, Bax and Caspase-3 were down-regulated after exercise. Bcl2 was upregulated after exercise (*P* < 0.05).

Western blot analysis showed that NR5A2, CYP7A1, and CYP8B1 were lowly expressed in metabolic syndrome, and their expression levels were up-regulated after exercise (*P* < 0.05) (Figure 10). IL1β, IL6 and TNFa are highly expressed in metabolic syndrome, and their expression levels are down-regulated after exercise. IL10 and SOD2 were lowly expressed in metabolic syndrome, and their expression levels were up-regulated after exercise (*P* < 0.05) (Figure 11). C/EBPb, FASN, SCD1, Bax and Caspase-3 were highly expressed in metabolic syndrome, and their expression levels were down-regulated after exercise. Bcl2 was low expressed in metabolic syndrome, and its expression level was upregulated after exercise (*P* < 0.05) (Figure 12).

## DISCUSSION

Metabolic syndrome can lead to lipid metabolism disorders, hypertension and diabetes, and even cardiovascular system lesions, affecting work and life. Although metabolic syndrome is not a disease, it is an important indicator of heart problem [13]. Exercise could enhance the adaptability of the organism [14]. The main results of this study were that NR5A2 expression was low in metabolic syndrome, and NR5A2 expression was up-regulated after exercise in patients with metabolic syndrome. After carrying out NR5A2 knockout or overexpression experiments, it was found that NR5A2 reduced the level of inflammation through CYP7A1/CYP8B1, and C/EBPβ-FASN-SCD1 reduced the level of apoptosis, thereby improving the metabolic syndrome.

NR5A2 is a member of the NR5A subfamily of four members (NR5A1-NR5A4) [15, 16]. NR5A2 is a key regulator of T cell proliferation and T cell-mediated immune responses [17]. It has an important anti-apoptotic function by regulating the transcription of cell cycle regulators, and its loss leads to basal and mitogen-induced cell death of T cells. Specific loss of NR5A2 in T cells strongly reduces proliferation induced by its activation, leading to impaired induction of T cell-regulated immune responses [18]. NR5A2 has important regulatory functions in different immune cells, directly regulating the transcription of Fas (CD95) ligands in cytotoxic T cells and controlling the production of proinflammatory cytokines in macrophages [19].

NR5A2 regulates intestinal glucocorticoid synthesis through transcription, glucose and lipid metabolism, and intestinal inflammation. It can also regulate immune cells through immunomodulator glucocorticoids [20]. NR5A2 plays an important role in differentiation of macrophage subsets and the development of anti-infective effector functions, as well as in cytokine-induced differentiation [21, 22]. NR5A2 plays an important role in pathophysiological processes and in the control of immune cell differentiation and function. NR5A2 has a constant nuclear localization and maintains a transcriptionally active conformation, but its activity is further regulated by coactivators and corepressors, post-translational modifications, and substances that interact with the ligand-binding capsule [23]. NR5A2 directly regulates the proliferation of intestinal crypt cells [24, 25].

NR5A2 activation has an effect on muscle metabolism. NR5A2 shifts muscle cells to a glucose preference state mainly by enhancing glucose uptake, and increased glucose utilization by muscle. NR5A2 stabilizes acinar properties, prevents inflammation, and is required for acinar recovery after injury. NR5A2 expression is down-regulated in metabolic syndrome [26]. Relevant literature reports that NR5A2 interacts with glucocorticoid receptors to regulate glucocorticoid resistance [23, 27]. Therefore, NR5A2 might affect metabolic syndrome.

NR5A2 can induce transcription in the absence of ligand binding and has an important role in steroidogenesis by regulating key steroidogenic enzymes [28]. NR5A2 is involved in a variety of biological processes such as hepatocyte differentiation, proliferation, liver cholesterol and glucose metabolism, relief of endoplasmic reticulum stress, intestinal glucocorticoid production, pancreatic development and acinar differentiation [29, 30]. Transcriptional regulation of NR5A2 links pancreatic differentiation and inflammation [31, 32]. Exercise can up-regulate the expression of NR5A2, and then regulate CYP7A1/CYP8B1 to reduce the level of inflammation, and regulate C/EBPβ-FASN-SCD1 to reduce the level of apoptosis. Therefore, it is speculated that exercise may improve metabolic syndrome by up-regulating the expression of NR5A2.

### Limitation

No clinical specimen experiments were performed to verify the role of NR5A2 in the metabolic syndrome.

In conclusion, NR5A2 is low expressed in MS and its expression level is upregulated when MS gets exercise. NR5A2 can be used as a potential target for exercise to improve metabolic syndrome.
